# Diseases of the Skin Elucidated by Anatomical Investigations

**Published:** 1852-03

**Authors:** 


### BIBLIOGRAPHICAL NOTICES.

*Die Hautkrankheiten durch anatomische Untersuchungen erläutert*, von Dr. GUSTAV SIMON, dirigirendem Artze am Charité-Krankenhause und Privatdocenten an der Universität zu Berlin. Mit 9 Kupfertafeln. Zweite vermehrte Auflagen. 8vo. S. 420. Berlin, 1851.

*Diseases of the Skin elucidated by anatomical Investigations.* By DR. GUSTAVUS SIMON, Directing Physician to the Charity Hospital, and *privatim docens* in the University of Berlin. *With nine Copperplates. Second enlarged Edition.* 8vo. pp. 420. Berlin, 1851.

*Manual of Diseases of the Skin, from the French of* MM. CAZENAVE and SCHEDEL. *With Notes and Additions*, by THOMAS H. BURGESS, M. D., Surgeon to the Blenheim street Dispensary for Diseases of the Skin, &c. *Second American edition, enlarged and corrected from the last French edition, with additional Notes*, by H. D. BULKLEY, M. D., Physician to the New York Hospital; Fellow of the College of Physicians and Surgeons, New York; Lecturer on Diseases of the Skin, &c. &c. 8vo. pp. 348. New York, 1852.

The works before us embody the results of the observations and reflections of two of the most eminent of living dermatologists. The Manual of MM. Cazenave and Schedel has been well known to us in consequence of its having been for a long time translated into English. The excellent work of Dr. Simon is less known here, but is considered to be classical in Germany; and the fact of the volume before us being the second edition of a work which treats only of the pathology of skin diseases, without a word being said of the treatment, exhibits the marked difference between our utilitarian views and those of our German brethren; for we fancy that no work on the mere pathology of such a class of diseases could experience a remunerative sale on this side of the Atlantic.

The bibliography of cutaneous affections is now rich. Admirable illustrations of the whole series have been published or are in course of publication in England and elsewhere; and there is none more worthy of commendation than that of Mr. Erasmus Wilson. The delineations are accurate and splendid; yet the price at which the fasciculi are published excludes them from the libraries of all except the more fortunate of the profession. To convey a correct idea of the diseases, the text ought always to be accompanied by such illustrations; but still a large amount of information can be brought home to the reader by accurate verbal descriptions. In this respect, indeed, pathological dermatology is in the same category with anatomy.

Dr. Simon first describes the normal skin, and then the morbid alterations which it experiences. Under the latter head he considers MORBID ALTERATIONS OF THE CUTIS AND EPIDERMIS; and, 1st, *Hypertrophies of the Skin and Epidermis*, Callositates, Clavus, Verrucæ, Ichthyosis and Elephantiasis Arabum; 2, *Atrophy;* and 3, *Hemorrhage from the skin;* followed by 4, *Inflammations or Phlegmasiæ of the Skin*—under which he classes Scarlatina, Morbilli, Variola, Varioloides, Varicellæ, Vaccina, Febris miliaris, Erysipelas, Urticaria, Erythema, Roseola, Rubeolæ, Lichen, Prurigo, Eczema, Herpes, Sudamina, Pemphigus, Rupia, Impetigo, Ecthyma, Pityriasis, Psoriasis, Pellagra, Furunculus, Anthrax, Ulcers and Gangrene of the skin. In the main features of this division there is an accordance between Dr. Simon, M. Rayer, Mr. Wilson and others. The next division investigates, 5, *Morbid new formations on the skin*—Cornua cutanea, Nævus mollusciformis or N. lipomatodes, congenital fatty tumors; Molluscum simplex, Condylomata, Nævus spilus; N. lenticularis; Ephelis; Melanosis of the skin and Lipoma, Cholesteatoma; Vascular tumors; Sarcoma of the skin; Osteoïd formations; encysted formations, and the various forms of cancer of the skin; Elephantiasis Græcorum, E. tuberculosa, E. non tuberculosa, and Lupus. 6, *Parasites of the skin*; including, first, parasitic animals; and secondly, parasitic plants.

The second great division is into MORBID ALTERATIONS OF THE CUTANEOUS GLANDS, HAIR FOLLICLES, HAIRS AND NAILS, under which he considers, *first*, alterations of the sudoriferous glands; *secondly*, alterations of the sebaceous glands and hair follicles; *thirdly*, alterations of the hairs; and *fourthly*, alterations of the nails.

It will thus be seen that the work of Dr. Simon enters more fully into the morbid condition of the skin and its appendages than the generality of treatises on cutaneous diseases; and as it is restricted to the pathological relations of the subjects on which it treats, it is full and generally satisfactory. The illustrations, which are the result of his own observations as well as those of the best microscopical anatomists of the day—Kölliker, Reichert, Krause, Henle, Bruns, Steinlin, Kohlrausch, Höfle, Fuchs, Gruby, Lebert, Robin and others—are highly elucidative of the anatomy of the skin and its appendages; of the different epithelial and other growths; of molluscous and condylomatous formations; of cancer of the skin; and of parasitic animals and plants; and, on the whole, the work contains a large amount of information to one who is engaged in the study of the essential nature of cutaneous affections.

The Manual of MM. Cazenave and Schedel—as already remarked—has been long before the medical public. In the year 1829, a translation was published in Philadelphia by the late Dr. R. E. Griffith; but the one before us is by Dr. Thomas H. Burgess of London, from the third edition of the French original, with notes and additions by the translator. It is the second American edition by Dr. H. D. Bulkley of New York, who has "enlarged and corrected" it from the last French edition, and appended additional notes. The original work embodied, as is well known, the substance of the views and experience in cutaneous diseases of M. Biett, who was one of the most distinguished dermatologists of France. "It was my text-book," says the English editor, "during the two seasons of my attendance at the Hospital of Saint Louis, under M. Biett, and since that period I have had increased opportunities in this country [England] of testing its value as a practical guide in the treatment of cutaneous affections. I can conscientiously say that I know of no other work of a similar kind, either in the English or any other language, which is preferable to that of MM. Cazenave and Schedel, nor one which would answer our present purpose so well. The clear and methodical manner in which the diseases are arranged, and the concise and simple style of the work, contrast favorably with the vague and obscure descriptions generally found in treatises on diseases of the skin."—*Preface to the English Edition.*

Equally encomiastic is the American editor. "Embodying as it does," he remarks, "the results of the long experience and accurate observations of M. Biett, so favorably known for his zeal and industry in the pursuit of this branch of our profession, and drawn up by pupils of this able teacher, who enjoyed the advantages of the same extensive field in which he himself studied, it may be safely recommended to both practitioners and students, as combining faithful and graphic descriptions of these diseases, and sound principles for their treatment. Having myself adopted it as a text-book in the study of them at the Hospital of St. Louis, (well known to be specially devoted to these diseases in Paris) and having also since used it in several successive courses of lectures on the subject in this city, I need hardly say that I fully coincide with Dr. Burgess in his opinion as to its merits." "The present edition," he adds, "has been carefully compared with the original, and numerous omissions supplied of passages which the translator thought proper to omit, but which it is thought, both in justice to the authors and to render the work more complete, ought to be restored. Some errors also have been corrected which doubtless escaped his observation. Notes have been added, consisting mostly of the Editor's experience in the treatment of these diseases during the last eleven or twelve years, both in dispensary and private practice, with references to other works and to the experience of those entitled to confidence." Thus much from the Preface to the first American edition. In the one prefixed to the edition before us Dr. Bulkley remarks: "Six additional years, during which the Editor has both studied and lectured upon these diseases, have furnished him with materials from his own experience and from that of others, the more important of which he has interwoven with the notes and references in the former edition; and he trusts he has been enabled in this way to enhance the value of the work, both to the student and the practitioner, without materially increasing its size."

All these gentlemen—it will be observed—followed the excellent instruction imparted on skin diseases at the Hôpital Saint Louis; and all may naturally be presumed to be partial to a manual, which embodies the observations and reflections of one so distinguished as M. Biett in the field of the profession which he so assiduously and successfully cultivated. We do not, however, think that their partiality has induced them to overestimate the work of MM. Cazenave and Schedel; we can, indeed, unhesitatingly endorse the encomiums they have passed upon it. The classification he adopts is what Dr. Simon calls the Willan-Bateman'sche Systeme, for which, in teaching, we are free to admit our preference, notwithstanding objections that may be urged against it—and what classification is free from them? It is on the basis of that of Plenck, "which," says MM. Cazenave and Schedel, "in the present state of our knowledge, cannot be surpassed in clearness and precision." And, in their last edition, they observe that it is in diagnosis that the classification of Willan exhibits its great advantages; and they add, that even if a natural classification of cutaneous diseases should at some future period be formed, that of Willan will always be retained for the purpose of diagnosis. Such is decidedly our own conviction; for, whilst we admit that the discrimination of the elementary lesions of the skin is not always easy, and that we may often remain in doubt as to which of the great divisions any special case may belong, it is liable to as few objections as any, and is decidedly more easy for the teacher and the student than any other. The classification of Biett, followed by MM. Cazenave and Schedel, like that of Willan, consists of eight orders—Exanthemata, Vesiculæ, Bullæ, Pustulæ, Papulæ, Squamæ, Tubercula and Maculæ; with the additional orders, Lupus, Pellagra, Malum Alepporum, Syphilida, Purpura, Elephantiasis Arabum and Cheloidea.

One form of difficulty in the diagnosis of a cutaneous disease, arises from the difficulty of appreciating the primary lesion as the elementary character may have disappeared, and given place to the secondary phenomena.

"The fluid of a vesicle"—say MM. Cazenave and Schedel—"may, for example, dry off and leave a small incrustation; a pustule may be converted into a scab, and the latter give way to an ulcer; hence it is necessary that we should study these secondary lesions, and know to what primary characters they correspond. Incrustations may succeed vesicles, vesicopustules, and papules; scabs occur in most pustular diseases, and ulceration may be a consequence of rupia, ecthyma, &c.

In cases like the foregoing, we must first ascertain the nature of the secondary lesion, then determine its corresponding primary element, and finally pursue the course just pointed out. For example, a patient comes to us with a disease of the skin, characterized by thick, rough, yellow scabs, which cover a large portion of the extremities, especially the legs, and when they fall off, expose superficial excoriations; the latter discharge purulent secretion, which dries up, and forms fresh scabs, these being the most characteristic features of the disease. Now it is easy enough to tell at once that this is a pustular affection, but not so easy to determine its species. The disease is evidently neither *variola* nor *vaccinia*; the pustules of *ecthyma* are large, isolated, and frequently covered by black, tenacious scabs, which end in ulceration; it is neither *acne* nor *mentagra*, the pustules of which rarely ever give rise to scabs, and are especially followed by chronic indurations. The only affections, then that remain are *impetigo* and *porrigo*, and we have merely to compare the character of these two species in order to decide. It is unnecessary to enumerate here the signs by which we know that the disease is not porrigo; it is therefore *impetigo*, and as the scabs are scattered irregularly over the limb, it is *impetigo sparsa*.

In the preceding cases we have supposed that there were no remains of the distinct elementary lesion, while, in the great majority of cases, on the contrary, some may always be found perfectly unchanged in the neighborhood of the affected part.

In some cases different elementary lesions occur in the same subject, but even here we always find some predominant form, of which the rest are but complications. However, it may happen that we cannot ascertain at once the true nature of the disease. This occurs in certain chronic affections, where the elementary character gradually disappears, and seems confounded in a different order of phenomena. Even here a sudden exacerbation of the disease, or a return to health, may develop its primary character. The general remarks which we have just made do not appply to those orders which are not characterized by special elementary lesions; but the latter are distinguished by phenomena which we cannot mistake; or even, when they assume, as in syphilis, the elementary forms of other cutaneous diseases, they present certain special appearances, which leave no doubt about their nature. Finally, we must neglect nothing which can assist us in our diagnosis of cutaneous diseases. Beside the elementary characters, there are many signs, as the seat, form, and color of the eruption, its progress, condition of the patient, &c., which strike the practical observer, and enable him often to dispense with details."—P. 24.

In regard to the treatment of chronic cutaneous diseases in general, it luckily happens, that the same indications are presented in all. Where undue irritation or inflammation of the skin is present, it has always to be combated by appropriate agents; but in such as have long continued, owing to a *vice* in the system of nutrition of the part, which has to be broken in upon, the same principles and the same agents for fulfilling these principles are generally indicated. There are but two modes on which we can act upon the affected tissues,—the one through remedial agents which may modify the blood, and through it the system of nutrition; the other through topical remedies, which are made to come into immediate contact with the diseased surface. Hence, the employment of such articles, as the oleum morrhuæ, and of the different preparations of iodine, of arsenic and of mercury, singly or combined, associated with various excitant ointments, is generally required. In the most inveterate cases, such an association is demanded; whilst in others topical remedies may be sufficient; but unless a proper system of discipline be enforced in regard to various affections of long standing, as lepra, psoriasis, trichonosis, &c., little effect will result from the application of any remedies. It may be laid down as a cardinal principle of management, that the cutaneous surface should be kept free from every concretion, by proper ablution, often repeated in the course of the day, and by the use of an appropriate unguent; and that by means of an oil silk covering, where this is practicable, the affected part should be maintained free from the irritating and desiccative influence of the air, so that the topical application may be kept in constant contact with the morbid surface; whilst the latter is preserved in the most favourable condition for the exercise of that recuperative action of the cells, without which all our remedial means must be fruitless; and if this course be persevered in, it is astonishing how successful will be the treatment of such cases; even after they have resisted the use of the same agents, less carefully and less systematically employed.

We would especially urge this desideratum in the treatment of chronic cutaneous diseases, from no little experience of its efficacy.

On every point connected with the management of cutaneous affections, the work of MM. Cazenave and Schedel, improved by the observations of Drs. Burgess and Bulkley, affords valuable information. The notes of Dr. Bulkley appear to be, in general, extremely pertinent. We observe, that he objects to the location of ichthyosis by MM. Cazenave and Schedel amongst the squamous affections as "misnamed as well as misplaced. There is nothing *scaly* about it." And we agree with him; but we are not so much in accordance with his view, that the name "*warty* disease" would be much more appropriate than that of fish-skin. "It is true"—he adds—"the cuticular appendages are not organized like true warts, and do not bleed on being removed; but otherwise their physical characters have a much greater affinity to those of warts than to the scales of fish." (Note, p. 240.) We should have objected less to the location of ichthyosis under "hypertrophies of the skin and epidermis," as has been done by Dr. Simon, (*Die Hautkrankheiten, s.* 42,); but we accord much more closely with the more recent views of Mr. Wilson on the pathology of this affection. "In the preceding edition of this work"—he remarks—"I regarded certain of these exodermal productions as hyperformations of epiderma, resulting from enlargement of the papillæ of the derma, while I retained others in the present group, under the designation of ichthyosis sebacea. I have since prosecuted my inquiries further into this subject, and have obtained clear evidence, that all the forms of ichthyosis are of the same nature; that they are, in fact, all concretions of altered sebaceous substance." (*On Diseases of the Skin*, 2d Amer. edit. p. 329, Philada., 1847.)

But we have exhausted our space, without having by any means exhausted the subject. All we can say, in conclusion, is, that the present edition of the work of MM. Cazenave and Schedel, enriched by its translator and annotators, is much more worthy of approbation than its predecessors; and may be consulted with eminent advantage, by every one who is desirous of being *à portée* with the existing condition of dermatological investigations, both as regards their pathology and therapeutics.

